# Human parainfluenza virus type 3 (HPIV3) induces production of IFNγ and RANTES in human nasal epithelial cells (HNECs)

**DOI:** 10.1186/s12950-015-0054-7

**Published:** 2015-02-21

**Authors:** Anna Lewandowska-Polak, Małgorzata Brauncajs, Edyta Paradowska, Marzanna Jarzębska, Marcin Kurowski, Sylwia Moskwa, Zbigniew J Leśnikowski, Marek L Kowalski

**Affiliations:** Department of Immunology, Rheumatology and Allergy, Chair of Clinical Immunology and Microbiology, Medical University of Lodz, Lodz, Poland; Department of Microbiology, Immunology and Laboratory Medicine, Chair of Clinical Immunology and Microbiology, Medical University of Lodz, Lodz, Poland; Laboratory of Molecular Virology and Biological Chemistry, Institute of Medical Biology, Polish Academy of Sciences, Lodz, Poland; Healthy Ageing Research Centre, Medical University of Lodz, Lodz, Poland

**Keywords:** PIV3, Human parainfluenza virus 3, Nasal epithelium, IFN-γ, RANTES, RPMI cells

## Abstract

**Background:**

Human parainfluenza virus type 3 (HPIV3), while infecting lower airway epithelial cells induces pneumonia and bronchiolitis in infants and children, and may lead to asthma exacerbations in children and adults. Respiratory viruses invading the airway epithelium activate innate immune response and induce inflammatory cytokine release contributing to the pathophysiology of upper and lower airway disorders. However, the effects of HPIV3 infection on nasal epithelial cells have not been well defined.

The aim of this study was to evaluate the effect of the HPIV3 infection on cultured human nasal epithelial cells (HNECs) and the release of interferon gamma and other cytokines.

**Methods:**

RPMI 2650, a human nasal epithelial cell line was cultured into confluence and was infected with HPIV3 (MOI of 0.1, 0.01 and 0.001). The protein release into supernatants and mRNA expression of selected cytokines were assessed 24, 48 and 72 h after infection. Cytokine concentrations in supernatants were measured by ELISA and expression of cytokine mRNA in RPMI 2650 cells confirmed by real time RT-PCR analysis.

**Results:**

HNECs infection with HPIV3 did not induce cytotoxicity for at least 48 hours, but significantly increased IFN-γ protein concentration in the cell supernatants at 24 h and 48 h post infection (by 387% and 485% respectively as compared to mock infected cells). At 24 h a significant increase in expression of mRNA for IFNγ was observed. RANTES protein concentration and mRNA expression were significantly increased at 72 h after infection (mean protein concentration: 3.5 ± 1.4 pg/mL for 0.001 MOI, 10.8 ± 4.6 pg/mL for 0.01 MOI and 61.5 ± 18.4 pg/mL for 0.1 MOI as compared to 2.4 ± 1.3 pg/mL for uninfected cells). No measurable concentrations of TNF-α, IL-10, TSLP, IL-8, GM-CSF or eotaxin, were detected in virus infected cells supernatants.

**Conclusions:**

HPIV3 effectively infects upper airway epithelial cells and the infection is associated with induction of IFN-γ and generation of RANTES.

## Introduction

Human parainfluenza viruses (HPIVs) which belong to the family *Paramyxoviridae* are enveloped, negative-strand RNA viruses and are common cause of acute airway respiratory illness in children and adults [1]. Out of four recognized serotypes PIV1 and PIV2 are leading causes of laryngotracheobronchitis in children, while PIV4 is a common respiratory pathogen similar to PIV3 in clinical presentation [2]. PIV3 is usually associated with bronchiolitis and pneumonia, and is among the most common cause of hospitalization in children [3-6]. In addition both in children and adults PIV3 infections have been implicated in exacerbation of bronchial asthma [7,8]. HPIV infection usually starts in the nose, and then spreads to the par nasal sinuses, Eustachian tubes, larynx, and eventually to the lower airways [9]. In upper airways of healthy adults the HPIV infection usually causes mild and transient symptoms (common cold) [3], but the role of HPIV in exacerbation of chronic upper airway diseases (rhino sinusitis) is largely unknown. In one study PIV3 was detected in HNECs from 88% of patients with post viral olfactory dysfunction as compared to 9% of control patients [10] suggesting potential involvement of PIV3 infection into the upper airway pathology.

Airway epithelium is the first line of defense during respiratory infection, and viral infection of lower airway epithelium with human respiratory viruses (rhinovirus, RSV or influenza) induce generation of a variety of cytokines, chemokines and interferons (including IFNs type I (α, β) or type III (λ) ), which are involved in the host defense and development of the airway inflammation [11-14]. However, few published studies evaluated parainfluenza virus interaction with the airway epithelium and little is known about the upper airway epithelial response to HPIVs infection [15,16].

Type I and type III, but not type II interferon (IFN-γ) are the predominant interferons induced by respiratory viruses in nasal epithelial cells [17]. IFN-γ is a cytokine with direct antiviral activity, capable of promoting NK cells and virus specific-T cells cytotoxicity, thus is considered an important molecule involved in antiviral host defense. Acting via its receptor IFN-γ activates hundreds of genes leading to pro-inflammatory effects by increasing antigen processing and presentation, and anti-inflammatory effects due to its apoptotic and anti-proliferative functions. IFN-γ may interact with the airway epithelium triggering specific receptors, and leading to reduction in STAT6 phosphorylation [18]. In the mouse model of asthma IFN-γ signaling through the airway epithelium inhibited mucus and chitinases production, and systemic eosinophilia independent of Th2 cell activation, suggesting its potential role in the modulation of asthmatic inflammation [19]. IFN-γ, has been considered to be mainly of lymphoid origin (produced mainly by T cells and natural killer cells) and relatively few studies investigated expression of IFN-γ by the airway epithelial cells [20].

Having in mind, the lack of experimental data on the HPIV interaction with the upper airway epithelial cells, and the paucity of information on the immune responses of the airway epithelium to HPIV3 infection, we employed human upper epithelial cell line (RPMI 2650) culture model to study virus-induced production of IFN-γ and pro-inflammatory cytokines.

## Materials and methods

### Cell and viral culture

Human nasal epithelial cells RPMI 2650 were obtained from the American Type Culture Collection (ATCC, Manassas, VA, USA). The cells were cultured in Eagle’s Minimum Essential Medium with Earle’s salt (Sigma, St. Louis, MO, USA), supplemented with 2 mM L-glutamine (Sigma), 10% fetal bovine serum (FBS, Invitrogen, Life Technologies, CA, USA) and penicillin 100 U/mL (Sigma), streptomycin 100 μg/mL (Sigma), amphotericin B 2.5 μg/mL (Sigma) at 37°C with 5% CO_2_ in humidified air. The culture medium was changed every 48 hours. The cells were seeded in 25 cm^2^ or 75 cm^2^ polystyrene cell-culture flasks (Becton Dickinson Ltd, Oxford, U.K) and were grown to 90% confluence. When cells reached confluence they were trypsinized with 0.1% trypsin-EDTA solution (Sigma) and cells were transferred to 24-well culture plates (BD Ltd) for further experiments.

HPIV type 3 was obtained from the American Type Culture Collection (ATCC, Manassas, VA, USA) and propagated in monkey kidney-derived LLC-MK2 cells (ATCC, Manassas, VA, USA). Viral stocks were prepared by infecting monolayer cultures of LLC-MK2 cells until cytopathic effects (CPE) were fully developed. Viral supernatant fluid was collected by centrifugation at 3000 rpm (960 × *g*) in an Eppendorf 5810R centrifuge (Eppendorf, Hamburg, Germany) equipped with an A-4-62 rotor (Eppendorf) for 10 min to clear cellular debris and frozen in aliquots at −80°C. All infections were performed on LLC-MK2 cell monolayers in serum-free medium. Virus titers were determined based on the 50% tissue culture infective dose (TCID50) assay by infecting LLC-MK2 cells with serial 10-fold dilutions of each virus stock. Infected cells were incubated at 37°C and CPE monitored on a daily basis. The TCID50 was calculated using the method of Reed and Muench. CPE was assessed by visual assessment and by assessment of the continuity of the monolayer after fixation in methanol (Sigma) and staining with 0.1% crystal violet (Sigma).

### Infection of cells with PIV3

RPMI 2650 cells cultured into confluence were placed in medium without serum or additives and then infected with PIV3 at multiplicities of infection (MOI) ranging from 0.001 to 1. After 1 h of incubation at room temperature, the inoculum was removed, and the cells were further cultured with MEM supplemented with 2% FCS. The cells or supernatants were harvested at relevant time point. The virus CPE and viability of infected cells were assessed 4, 8, 24, 48 and 72 h after infection.

### Viability assay

Cell viability was assessed using the MTT assay. The RPMI 2650 cells were trypsinized, replated in 96-well plates and allowed to attach overnight. Fresh medium containing PIV3 was added to the cells. On specified time points, the culture media was removed, and MTT [thiazolyl blue tetrazolium bromide, 3-(4, 5-dimethylthiazol-2-yl)-2, 5-diphenyltetrazolium bromide] (Sigma) was added to each well and the plates were incubated at 37°C for 3 h. The reaction was stopped and after removing the medium, DMSO was added to solubilize the blue-colored tetrazolium. The absorbance at 570 nm was determined using an ELISA plate reader. Each assay point was performed in triplicate and the assays were performed on at least three separate occasions with similar results.

### Measurement of mediators

TNF-α, IL-10, TSLP, IL-8, RANTES, eotaxin, GM-CSF and IFN-γ levels were measured in cell supernatants 24, 48 and 72 h after infection with HPIV3 (MOI of 0.001, 0.01 and 0.1) by commercially available ELISA kits (R&D Systems, Minneapolis, MN, USA) according to the manufacturer’s specifications.

The sensitivity of the immunoassays was as follows: RANTES 3 pg/ml; IL-10 3.9 pg/mL, TSLP 9.87 pg/mL, IL-8 1.5-7.5 pg/mL; eotaxin 5 pg/mL; GM-CSF 3 pg/mL, IFN-γ 1.8 pg/mL.

### Real time PCR

Expression of cytokine mRNA in RPMI 2650 cells was evaluated after reverse transcription with real-time polymerase chain reactions. Total cellular RNA was extracted from epithelial cells using RNeasy mini kit (Qiagen, Limburg, Holand) and reverse transcription was performed with Omniscript RT kit (Qiagen). Real-time fluorescent detection PCR product analysis was performed by using iQ SYBR-Green Supermix (Bio-Rad, Hercules, CA, USA) according to instrument recommendations (StepOnePlus Real Time PCR System, Applied Biosystems, Carlsbad, California). PCR amplification of cDNA was performed in a reaction mixture containing 1 μL cDNA, 1 μL forward and 1 μL reverse primers, 7 μL H_2_O and 10 μL iQ SYBR GreenSupermix in a total volume of 20 μL. Reaction conditions were as follows: 95°C for 5 min, followed by 35 cycles of 95°C for 30 sec, 35 cycles of different temperatures for 1 min and 35 cycles of 72°C for 1 min. Annealing temperatures were as follows: 60°C for IFN-γ and 61°C for RANTES. Relative quantification of different transcripts was determined with the 2^−ΔΔCT^ method using β-actin as an endogenous control.

Primers specific for RANTES and IFN-γ were purchased from Eurogentec (Eurogentec, Liege, Belgium). The sequences of the primers were as follows: RANTES: Forward 5′- CGC TGT CAT CCT CAT TGC TA -3′, Reverse 5′-GAG CAC TTG CCA CTG GTG TA-3′; IFN-γ: Forward 5′-ATA TTG CAG GCA GGA CAA CC-3′, Reverse 5′-TCA TCC AAG TGA TGG CTG AA-3′; β-actin: Forward 5′- AGA AGG ATT CCT ATG TGG GCG-3′, Reverse 5′- CAT GTC GTC CCA GTT GGT GAC-3′.

### Statistical data analysis

Data are presented as means and standard errors of the means. Statistical analyses of the mediator concentrations were performed using Kruskal-Wallis ANOVA, followed by Wilcoxon matched-pairs signed-rank test. Alternatively, Mann–Whitney U test was performed. Subsequent post hoc analysis was conducted using the Bonferroni-adjusted α method. All statistical analyses were performed using Statistica version 10 (StatSoft Inc.). Values of p lower than 0.05 were considered as statistically significant.

### Ethics statement

The study was approved by ethics committee of the Medical University of Lodz (RNN/121/12/KE 19/06/2012).

## Results

### HPIV3 cytotoxicity for RPMI 2650 cells

**Figure 1:**
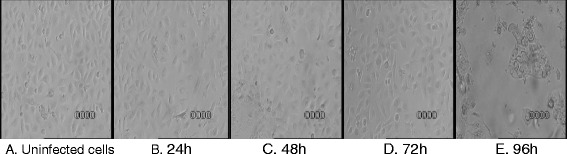
**Confluent RPMI 2650 cells monolayers under the light microscope (100x). A**. non-infected; **B**, **C**, **D**, **E** – infected with PIV3 at an MOI of 0.1 at different time-points.

**Figure 2:**
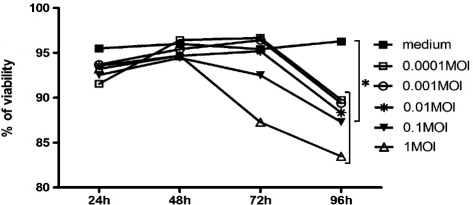
**Cell viability changes of PIV3-infected RPMI 2650 cells.** Cells were seeded into 24-well culture plates and infected with PIV3 at different MOI for indicated hours. Cell viability was detected by MTT assay. (n = 10).

To determine whether HPIV3 replication in RPMI 2650 cells had any cytotoxic or cytopathic effect, cell cultures were infected with the virus and observed using an inverted microscope for the development of cytopathic effect up to 96 h post inoculation (p.i.). Any cytopathic effect (rounding, bridging, cell lysis, and syncytium formation) was observed after 72 h and at the highest MOI of 1. (Figure 1) When monolayers were stained with crystal violet, no disruption of the cell layer could be observed before 72 h. In order to assess the effect of virus infection on cells’ viability, the culture media was removed at specified time points, and MTT was added to each well. At 24, 48 and 72 h there was no difference in cell viability between the infected (MOI of 0.0001, 0.001, 0.01, and 0.1) and uninfected cells (Figure 2). Decreased cell viability occurred at 72 h after virus infection at 1 MOI and this effect was pronounced 96 h after infection. Based on these experiments optimal MOI and time points were chosen for further experiments.

### Cytokine release and expression in nasal epithelial cells in response to HPIV3 infection

**Figure 3:**
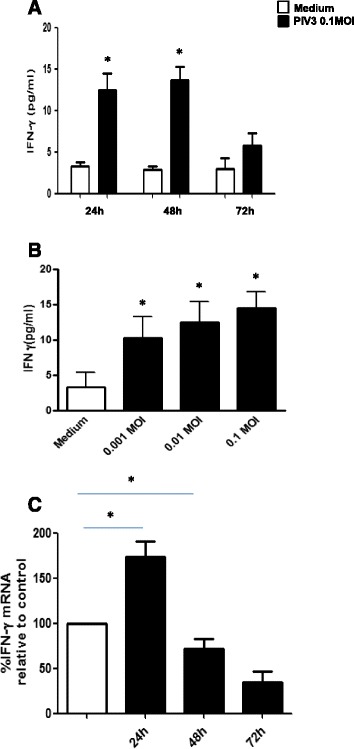
PIV-3 induced IFN-γ protein release and mRNA expression in RPMI 2650 cells (n = 8; the results are expressed as mean +/− SEM; * p < 0.05). A. Time course of PIV-3 (0.1 MOI) induced IFN-γ protein release. **B**. Effect of increasing PIV-3 MOI on IFN-γ protein release at 24 h p.i. **C**. Time course of PIV3 (0.1MOI) induced IFN-γ mRNA expression.

IFN-γ protein was detected in non-infected cell supernatants at 24 h (mean concentration 3.2 ± 1.2 pg/mL), 48 h (mean 2.8 ± 0.9 pg/mL) and 72 h (mean 2.9 ± 1.9 pg/mL). PIV3 infection significantly increased in IFN-γ protein level at 24 (by mean 387%) and 48 h (by mean 485%), post infection but the level significantly decreased at 72 h. Figure 3A The increase in the IFN-γ protein concentration at 24 h was significant for all three MOI as compared to mock infected cells (for MOI 0,001 - mean 10.2 ± 3.1 pg/mL, MOI 0,01 - mean 12.4 ± 2.9 pg/mL and MOI 0,1- mean 14,8 ± 2.3 pg/mL) Figure 3B.

IFN-γ mRNA expression increased significantly at 24 h by 174% over non-infected control and then decreased as compared to control by 28% and 66% at 48 h and 72 h after infection respectively. Figure 3C.

Out of 7 cytokines measured in HNECs supernatants after HPIV3 infection only RANTES protein was detectable. No measurable concentrations of TNF-α, IL-10, IL-8, exotaxin, GM-CSF and TSLP were detected in both uninfected and virus-infected cells supernatants.

**Figure 4:**
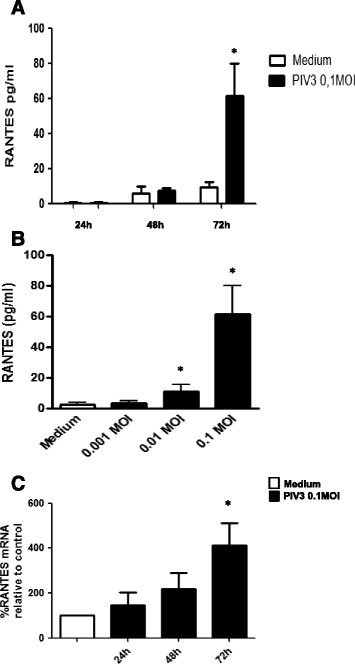
PIV-3 induced RANTES protein release and mRNA expression in RPMI 2650 cells (n = 8; the results are expressed as mean +/− SEM; * p < 0.05). A. Time course of PIV-3 (0.1MOI) induced RANTES protein release. **B**. Effect of increasing PIV-3 MOI on RANTES protein release at 72 h p.i. **C**. Time course of PIV3 (0.1MOI) induced RANTES mRNA expression.

RANTES protein was undetectable in supernatants collected at 24 h, but was detected at 48 h in supernatants from uninfected and PIV3 infected cells (0.1MOI) (mean concentration 6.1 ± 3.2 pg/mL and 7.5 ± 2.1 pg/mL) and significantly increased at 72 h p.i. (mean concentration 61.5 ± 18.4 pg/mL as compared to 9.3 ± 2.8 pg/mL in non-infected cells). Figure 4A The increase in RANTES protein release at 72 h was virus dose-dependent. Figure 4B. RANTES mRNA expression slightly increased already at 24 h (mean 15% increase over non-infected control) and at 48 h (mean 51% increase), but was significantly augmented (mean 184% increase) at 72 h Figure 4C.

## Discussion

Our study demonstrated for the first time, that human airway epithelial cells may generate IFN-γ in response to virus infection. Generation of IFN-γ by epithelial cells tended to be virus dose-dependent and the maximal signal for IFN-γ mRNA expression was observed already at 24 h after infection together with significant increase in IFN-γ protein concentration in the cell supernatant. Interestingly, IFN-γ protein concentration decreased to baseline level at 72 h post infection in parallel to decreased IFN-γ mRNA expression. At 72 h post infection the cells maintained full viability and RANTES expression reached the highest value, indicating that the IFN-γ generation was a truly transient phenomenon. Our observations contrast with previous study that failed to detect IFN-γ mRNA expression or IFN-γ protein secretion in primary human airway epithelial cells in response to rhinovirus (RV16) infection [14]. However, IFN-γ was shown to be expressed in the alveolar cell line A549 after bacterial infections: IFN-γ mRNA expression was detected in cells infected with *Mycoplasma pneumoniae* and *Chlamydia pneumoniae* [21,22] and release of high concentrations of IFN-γ was shown in A549 cells infected with *Mycobacterium tuberculosis* [20]*.* These observations suggest, that IFN-γ generation by airway epithelial cells may depend on the infecting pathogen and/or on the epithelial cell line tested. In an ex vivo study, IFN-γ expression in PBMC of infants varied significantly depending on the nature of the viral pathogen (parainfluenza, rhinovirus, adenovirus or RSV) and severity of clinical illness [23]. Differential capacity of respiratory viruses to generate IFN-γ response in the airway epithelial cell may be important to determine their ability to promote Th1 immune response, which especially in infants may have consequences for development of allergic sensitization and asthma later in life. While these observations require to be confirmed in the primary airway epithelial cells, they may have potential implications for our understanding of the pathophysiology of PIV-3 induced respiratory infection. IFN-γ induces genes promoting antiviral mechanisms, which include leukocyte recruitment, antigen processing and presentation, cell proliferation and apoptosis, leading to limitation of the viral infection and its consequences [24]. IFN-γ actions through the airway epithelium has been shown to inhibit eosinophil generation in the bone marrow, limiting airway obstruction and inflammation in the animal model of asthma [19]. Thus, induced by virus generation of IFN-γ in epithelial cells must be considered as a component of natural host defense counteracting induction of pro inflammatory mediators and leading to limitation of the tissue destruction. IFN-γ may act in concert with type I (IFN-α, IFN-β) and type III (IFN-λ) interferons, which are generated in response to viral infections in upper and lower airway epithelial cells and may limit virus-induced injury [17,25].

On the other hand it has been documented, that local production of IFN-γ as well as other proinflammatory cytokines (CCL3, CCL 11) in the lungs of mice infected with murine parainfluenza (Sendai Virus) correlated with severity of the lung disease [26]. In cotton rat model of Human Parainfluenza 3 laryngotracheitis IFN-γ mRNA expression in laryngeal tissues was increased by infection and corticosteroid treatment, which reduced the extent of lesions, led to a measurable reduction of IFNγ expression [27]. Accordingly in children with croup parenteral dexamethasone or inhaled budesonide are used in the early treatment of the diseases, and effectiveness of GCS has been associated with decreased IFN-γ mRNA expression [28]. Thus, further studies with specific inhibitors of IFN-γ have to be carried out to elucidate if virus induced IFN-γ generation by epithelial cells has pro or anti-inflammatory function.

HPIV3 has been shown to exacerbate preexisting asthma presumably by setting off the inflammatory cascade with generation of other than IFN-γ mediators and cytokines [7,8,29]. In human upper airway epithelial cell line infected with HPIV3 out of 7 cytokines measured only RANTES could be detected in the supernatants. A significant increase in RANTES protein was observed at 72 h, when IFN-γ concentration was already decreased. The induction of high RANTES levels in PIV3 infected of upper airway epithelial cells is consistent with other studies on bronchial epithelial cell monolayers infected with HPIVs and other viruses from *Paramyxoviridae* family [15,30,31]. In human ciliated airway epithelium infected with PIV3 RANTES concentrations increased gradually from day 2 through day 5 post infection [15]. Increased RANTES secretion was also found after RSV infection in bronchial epithelial cells [30,31] and elevated RANTES levels were reported in nasopharyngeal secretions from RSV-positive children [32]. Local production of chemokines has been also observed in children with PIV-positive airway respiratory infections: PIV-infected patients had higher nasal wash concentrations of chemokines such as RANTES/CCL5, IL-8/CXCL8, MIP-1 α/CCL3, MIP-1 β/CCL4, CXCL9 and CXCL10 as compared to uninfected control patients [33]. Production of pro-inflammatory chemokines by HPIV3 infected airway epithelial cells may contribute to development of respiratory symptoms in infected healthy individuals, but may also contribute to the exaggerated host inflammatory response associated with virus - induced exacerbations of chronic inflammatory airways diseases .

Although we were not able to detect any measurable concentrations of TNF-α, IL-10, IL-8, exotaxin, GM-CSF and TSLP in either uninfected and virus-infected cells supernatants our data cannot be directly referred to in vivo studies. For example, the release of cytokines and chemokines from airway epithelial cells is strongly affected by the presence of T lymphocytes, which may be responsible for less cytokine generation in isolated cell line model [34]. Furthermore, cytokine production in response to virus infection is both virus and cell type specific and even the same virus may evoke various cytokine response in upper and upper and lower primary human epithelial cells [35]. Thus, is also possible that the lack of production of some proinflammatory cytokines in our model results either from not sufficient activation of cells by PIV3 or reflect specific type o response to HPIV3 virus in RPMI cell line.

RPMI 2650 cell line used in this study have been shown to closely resemble normal human upper airway epithelium with respect to its karyotype, cytokeratin expression and the presence of mucoid material on the cell surface, and were previously used to study interactions of the airway epithelium with cytokines and allergens [36-38]. This is the first study documenting *in vitro* infection of human nasal epithelial cells (RPMI 2650 line) with HPIV3, and its association with the release of cytokines; in all previous studies only HPIV infections of lower airway epithelium have been examined [15,16,39]. In our model HNECs infected with HPIV3 did not show apparent cytopathology for up to 92 h after infection, and cells viability was not changed up to 48 h, and at 72 h only with the highest virus titres was significantly decreased. These results are consistent with observations of Zhang et al [39,40]. who during the lower airway epithelial cells culture, grown at an air-liquid interface, observed little PIV3 virus cytotoxicity.

In conclusion we have demonstrated that HPIV3 effectively infects upper airway epithelial cells and the infection is associated with induction of interferon gamma. Further investigations involving primary human cells and IFN-γ inhibitors may allow for understanding the role of the epithelium derived IFN-γ in the pathophysiology of HPIV3 induced and/or exacerbated respiratory disorders.

## Abbreviations

HPIVs: Human parainfluenza viruses
PIV3: Parainfluenza virus type 3
HPIV3: Human parainfluenza virus type 3
RSV: Respiratory syncytial virus
HNECs: Human nasal epithelial cells
